# Nicotinamide Riboside Supplementation Protects Against Heat-Induced Skeletal Muscle Injury in Female Mice

**DOI:** 10.3390/muscles5020044

**Published:** 2026-06-15

**Authors:** Yifan Chen, Tianzheng Yu

**Affiliations:** 1 Department of Military and Emergency Medicine, F. Edward Hébert School of Medicine, Uniformed Services University, Bethesda, MD 20814, USA; tianzheng.yu.ctr@usuhs.edu; 2 The Henry M Jackson Foundation for the Advancement of Military Medicine, Inc., Bethesda, MD 20817, USA

**Keywords:** heat stress, hyperthermia, niacin, skeletal muscle, ATP, gender

## Abstract

Micronutrients are essential for optimal muscle metabolic function. We previously showed that heat-induced skeletal muscle injury is associated with depletion of nicotinamide adenine dinucleotide (NAD^+^) and magnesium (Mg^2+^), and boosting NAD^+^ abundance with the precursor nicotinamide riboside (NR) improves skeletal muscle integrity against heat stress in male mice. In this study, we hypothesized that NR supplementation would prevent heat-induced skeletal muscle injury in female mice. Female 6-week-old C57BL/6J mice were orally administered vehicle or NR (185 mg/kg body weight) daily for 10 days. Subsequently, they underwent a single sham or heat exposure experiment. No significant differences in muscle NAD^+^ content were observed between vehicle and NR groups or between sham and heat groups. Heat groups showed significantly lower muscle Mg^2+^ levels compared to sham groups. In vehicle groups, heat exposure caused significant inflammation, oxidative stress, mitochondrial impairment, and apoptosis in skeletal muscle compared to the sham condition. NR treatment significantly reduced these alterations. While neither heat exposure nor NR affected muscle NAD^+^ homeostasis, the protective effects of NR on skeletal muscle against heat stress were similar to those observed in male mice. Together, our results demonstrate the preventive effect of NR on muscle heat injury in female mice. This effect is associated with anti-inflammatory and antioxidative activities, mitochondrial protection, and anti-apoptosis without NAD^+^ homeostatic alterations.

## 1. Introduction

Heat stress can cause damage to various organs [1,2,3,4]. Micronutrients are essential for maintaining metabolic homeostasis and cell survival during heat stress [5,6]. We previously showed that heat-induced skeletal muscle apoptosis in mice is associated with inflammation, oxidative stress, and mitochondrial impairment [4]. Research surrounding nutritional and dietary supplements for preventing heat stress [7] or heat-related organ injury [8] has received considerable attention. Although preliminary evidence has shown some promising candidates, their beneficial effects have not been consistent. There is a lack of information on how heat stress affects nutrient homeostasis in tissues and organs.

We recently reported that heat-induced skeletal muscle injury in male mice is associated with reduced levels of nicotinamide adenine dinucleotide (NAD^+^) and magnesium (Mg^2+^) [9]. Both NAD^+^ and Mg^2+^ are crucial coenzymes or cofactors that regulate cellular redox homeostasis and energy metabolism. Heat stress is known to disrupt cellular redox balance, and previous studies, including ours, have demonstrated that heat exposure induces oxidative stress in cells [4,9,10,11] and tissues [9,11,12]. Importantly, our research showed that supplementing male mice with nicotinamide riboside (NR), an NAD^+^ precursor, mitigated heat-induced oxidative stress and apoptosis in skeletal muscle [9]. Interestingly, these beneficial effects of NR were associated with the restoration of tissue NAD^+^ levels but not Mg^2+^ levels. This suggests that the disruption of Mg^2+^ homeostasis is more likely a consequence of heat injury rather than its cause. Furthermore, we previously reported that female mice had a significantly lower hyperthermic response to heat exposure compared to males [13,14], despite no differences in skeletal muscle injury-related changes between the sexes [14]. It remains unclear how heat stress impacts NAD^+^ and Mg^2+^ homeostasis and whether NR provides similar anti-heat stress benefits in female mice.

In this study, we tested the hypothesis that NR supplementation would provide a protective effect against heat-induced skeletal muscle injury in female mice. Specifically, we examined NAD(H) levels in the skeletal muscle and Mg^2+^ and Ca^2+^ levels in the blood and different organs of female mice with or without NR supplementation. We assessed inflammation, oxidative stress, apoptosis, and mitochondrial impairment in the skeletal muscle. Herein, we show that NR protected the skeletal muscle against heat injury. Neither heat stress nor NR altered muscle NAD^+^ homeostasis.

## 2. Materials and Methods

### 2.1. Animals

Female C57BL/6J mice, 6 weeks of age, were purchased from Jackson Laboratories (Bar Harbor, ME, USA). The mice were housed at the Uniformed Services University (USU) Department of Laboratory Animal Resources in an enriched controlled environment (22 °C, 12:12 h light-dark cycle) and fed a standard mouse chow (LabDiet 5V75). All procedures were approved by the USU Institutional Animal Care and Use Committee (Protocol MEM-22-059) and strictly adhered to Federal regulations for animal protection in research. A temperature-sensing transponder (BioTherm13, Biomark Inc., Boise, ID, USA) was surgically implanted into the abdominal cavity of all mice while under anesthesia. Following a minimum recovery period of one week, mice were randomly assigned to receive daily treatment for 10 days by gavage with either vehicle (water) or nicotinamide riboside (NR) at a dose of 185 mg/kg body weight [9]. The treated mice were then divided into two subgroups (6 mice/group): heat and control. Control mice were kept at room temperature (sham), while heat-exposed mice underwent a single heat stress test. Upon completion of the experiments, the animals were euthanized, and blood was immediately collected via cardiocentesis under anesthesia before tissue samples were harvested.

#### 2.1.1. Heat Exposure

Heat exposure experiments were performed in an environmental chamber (Model 3950, Thermo Forma, Marietta, OH, USA). All mice were acclimatized overnight within the chamber at room temperature (22 °C). The following morning, the experiment initiated, with food and water removal, and stable core body temperature (Tc) baseline measurements were acquired. The environmental chamber’s heating element was then activated, maintaining a set temperature of 39.5 °C for a duration of 180 min as described previously [4]. Throughout the procedure, the body core temperature of each mouse was continuously monitored and recorded.

#### 2.1.2. Enzyme-Linked Immunosorbent Assays

To quantify inflammatory cytokines and oxidative stress markers, concentrations of interleukin 6 (IL-6), tumor necrosis factor alpha (TNFα), and thiobarbituric acid reactive substances (TBARS) were assayed in gastrocnemius muscle tissue. The measurements utilized commercially available enzyme-linked immunosorbent assay (ELISA) kits following the manufacturers’ protocols: Mouse IL-6 ELISA (Abcam, Waltham, MA, USA, #ab100713), Mouse TNF Alpha ELISA (Abcam, #ab108910), and TBARS Assay (Cayman Chemical, Ann Arbor, MI, USA, #10009055) [15].

#### 2.1.3. Measurement of Reactive Oxygen Species (ROS) and Apoptosis

Reactive oxygen species (ROS) and apoptosis were detected in fresh flexor digitorum brevis (FDB) muscle fibers. Briefly, dihydroethidium (DHE, 5 µM, Thermo Fisher Scientific, Waltham, MA, USA) was used to detect ROS, while CellEvent Caspase-3/7 Green reagent (Thermo Fisher Scientific, #R37111) was used to detect apoptosis. DHE fluorescence was quantified at an excitation/emission wavelength of 555/613 nm, and caspase 3/7 fluorescence was quantified at 480/535 nm. Fluorescence images were viewed and acquired using a Nikon Eclipse Ti epifluorescence microscope (10× objective) (Melville, NY, USA) equipped with a digital camera. Quantification of the fluorescence signals for both ROS and apoptosis was performed using ImageJ software (version 1.51, NIH) as described previously [9].

#### 2.1.4. Mitochondrial Morphology and Membrane Potential

Single myofibers were prepared from fresh FDB muscles via a two-step collagenase/dispase enzymatic digestion protocol, as we described previously [14]. Mitochondrial morphology was assessed using the fluorescence dye MitoTracker Red CMXRos (100 nM, Thermo Fisher Scientific). For quantifying mitochondrial membrane potential (ΔΨm), the cationic fluorescent probe tetramethylrhodamine ethyl ester (TMRE, 100 nM, Thermo Fisher Scientific) was used. Prior to imaging, muscle fibers were incubated with the respective fluorescent dyes for 15 min protected from lights. Fluorescence images were captured using a Nikon Eclipse Ti epifluorescence microscope, equipped with a 10× objective and a digital camera, to view and acquire data. For morphology, mitochondrial fragmentation was quantified by calculating the percentage of muscle fibers that contained 10 or more fragmented (short and punctate) mitochondria per fiber. Approximately 30 FDB fibers were randomly selected from 6 to 10 random fields for each mouse, for comprehensive analysis of both fragmentation and membrane potential. The TMRE fluorescence intensity, which serves as a quantitative measure for ΔΨm, was determined using NIH ImageJ software.

#### 2.1.5. ATP Assay

The quantification of ATP content was performed using the ATP Assay Kit (Abcam, ab83355), by following the manufacturer’s protocol. Briefly, gastrocnemius muscle samples were rinsed with cold phosphate-buffered saline (PBS) and then homogenized within the provided ATP assay buffer. The resulting homogenates were then centrifuged at 13,000× *g* for 3 min at 4 °C, and the supernatant fraction was subsequently isolated. For measurement, 50 µL of the tissue lysate was added with the reaction formulation in a 96-well microplate. This mixture was protected from light and incubated for 30 min at room temperature. Final ATP concentrations were measured spectrophotometrically at 570 nm using the Bio-Rad iMark™ Microplate Absorbance Reader (Bio-Rad Laboratories, Inc., Hercules, CA, USA) for Life Science and standardized against the total protein content of each sample.

#### 2.1.6. Measurement of NAD(H)

The total NAD (NADt) and NADH levels were assayed in snap-frozen gastrocnemius muscle (approximately 10 mg) utilizing the NAD/NADH Assay Kit (ab65348, Abcam), following a protocol detailed in our prior work [9]. Briefly, tissue samples were homogenized in ice-cold extraction buffer, followed by centrifugation for 5 min at 4 °C to collect the supernatants. To measure NADH, 100 µL portions of the extracted sample were incubated in a 60 °C water bath for 30 min, to degrade NAD^+^ while retaining NADH. Both the total NAD and NADH fractions were then added with the NAD Cycling Enzyme Mix and NADH Developer in a 96-well microplate. Following an incubation period of 1 h at room temperature, the absorbance at 450 nm was recorded using the Bio-Rad iMark™ Microplate Absorbance Reader. NAD^+^ and NADH concentrations were calculated according to the kit protocol: the concentration of NAD^+^ was indirectly derived by subtracting the measured NADH concentration from the measured total NAD concentration (NAD^+^ = NADt − NADH). NAD^+^ and NADH contents were finally normalized to the total tissue protein.

#### 2.1.7. Measurement of Ca^2+^ and Mg^2+^

Magnesium and calcium levels in gastrocnemius muscle, jejunum and kidney and plasma were quantified using the Magnesium Assay Kit (ab102506) and the Calcium Assay Kit (ab102505), both purchased from Abcam (Cambridge, MA, USA), by following the manufacturer’s instructions. Briefly, tissue samples were homogenized in the Magnesium or Calcium Assay Buffer coming with each kit. The resulted homogenates were centrifuged at 13,000× *g* at 4 °C for 10 min, and the supernatants were collected, along with plasma samples, and then diluted within the linear range of the assays. For the magnesium assay, samples were incubated with the Magnesium Reaction Mix at room temperature for 10 min, and absorbance was measured at 450 nm. For the calcium assay, samples were incubated with the Calcium Assay Reagents at room temperature for 5–10 min, protected from light, and the absorbance at 575 nm was measured using a Bio-Rad microplate reader. The total protein levels in tissue homogenates were determined using a BCA assay, and magnesium or calcium levels were normalized to the protein concentration (nmol/mg protein).

For calcium imaging, fresh FDB fibers were incubated with one of the membrane-permeable fluorescent calcium probers, Fluo-4-AM or Rhod-2-AM (Thermo Fisher Scientific) [9]. The muscle fibers were loaded with 1 μM Fluo-4-AM along with 0.02% Pluronic F-127 dissolved in HEPES buffer for 30 min for the detection of cytosolic Ca^2+^ or loaded with Rhod-2-AM (5 μM) at room temperature for 15 min for the detection of mitochondrial Ca^2+^. The samples were then washed with indicator-free HEPES buffer and incubated for 30 min to allow for de-esterification before experiments. The membrane-permeable Mag-Fluo-4 AM (Thermo Fisher Scientific) was used for cytosolic and mitochondrial magnesium imaging. We measured the mitochondrial Mg^2+^ levels using a membrane permeabilization protocol. First, FDB fibers were incubated with 1 μM Mag-Fluo-4 AM. The fibers were then permeabilized with 20 μg/mL digitonin in an intracellular-like medium (ICM, containing 120 mM KCl, 10 mM NaCl, 1 mM KH2PO4, 20 mM HEPES-Tris, 2 mM MgATP, and pH = 7.2) for 3 min, and subsequently, they were washed out with a detergent digitonin before experiments. The fluorescence signals were measured at the excitation/emission wavelengths: Fluo-4 494/506 nm, Rhod-2 552/581 nm, Mag-Fluo-4 AM 493/517 nm, and MitoTracker Red 579/599 nm. Images were obtained using a 10× objective and quantified using the ImageJ software (NIH).

### 2.2. Statistical Analysis

Statistical evaluations were performed using GraphPad Prism 10.4. A Two-way ANOVA was implemented to assess the variance across groups, with subsequent post hoc analysis performed using Tukey’s test. All quantitative data are reported as the mean ± SD, and a value of *p* < 0.05 was designated as the threshold for statistical significance.

## 3. Results

### 3.1. NR Reduces Inflammation and Oxidative Stress in Skeletal Muscle

Following 10 days of dietary treatments, there were no significant differences in body weight between the vehicle and NR groups (18.3 ± 1.7 g vs. 17.8 ± 0.9 g, n = 12 per group, *p* < 0.001). Subsequently, six mice from each group underwent a heat exposure test. There were no significant differences between the two groups in baseline body core temperature (Tc) or in peak Tc during heat exposure (Table 1).

**Figure 1:**
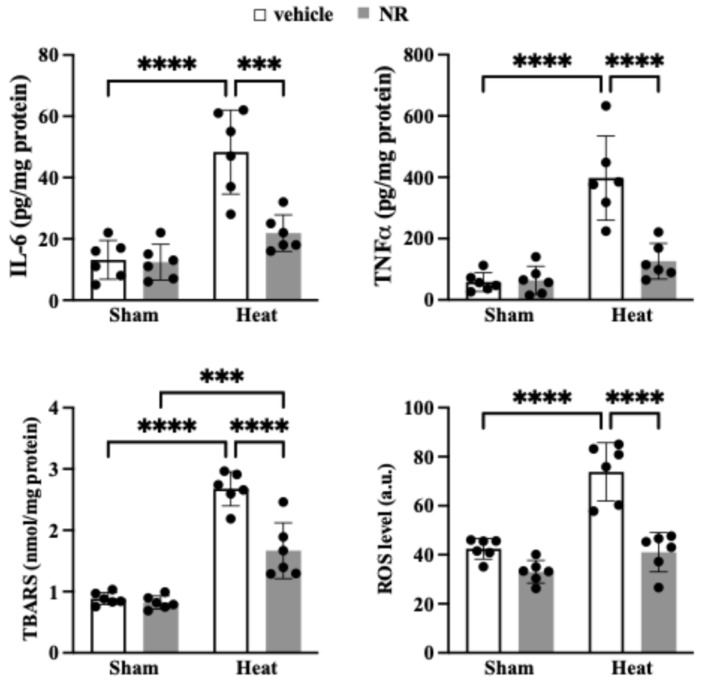
Inflammatory and oxidative stress markers in skeletal muscles. IL-6, TNFα and TBARS were measured in gastrocnemius muscle using the corresponding ELISA kits. ROS levels were quantified in FDB muscle based on DHE fluorescence (Figure S1). Data are shown as means ± SD (n = 6 mice per group). *** *p* < 0.001, and **** *p* < 0.0001.

Our previous studies have shown that the protective effect of NR against mouse organ injury was associated with the inhibition of inflammation [15] and oxidative stress [9]. We measured the proinflammatory cytokines IL-6 and TNFα and the lipid oxidation products TBARS in the gastrocnemius muscle. Compared with the corresponding sham mice, the levels of IL-6, TNFα and TBARS were significantly elevated in heat-exposed mice treated with vehicle (*p* < 0.001–0.0001, n = 6 per group, Figure 1). NR treatment prevented or reduced these increases. We further examined the ROS levels in FDB muscles with DHE fluorescence microscopy (Figure 1 and Figure S1). Heat exposure significantly increased the ROS levels, and NR treatment abolished this effect.

### 3.2. NR Reduces Mitochondrial Impairment and Apoptosis in Skeletal Muscle

**Figure 2:**
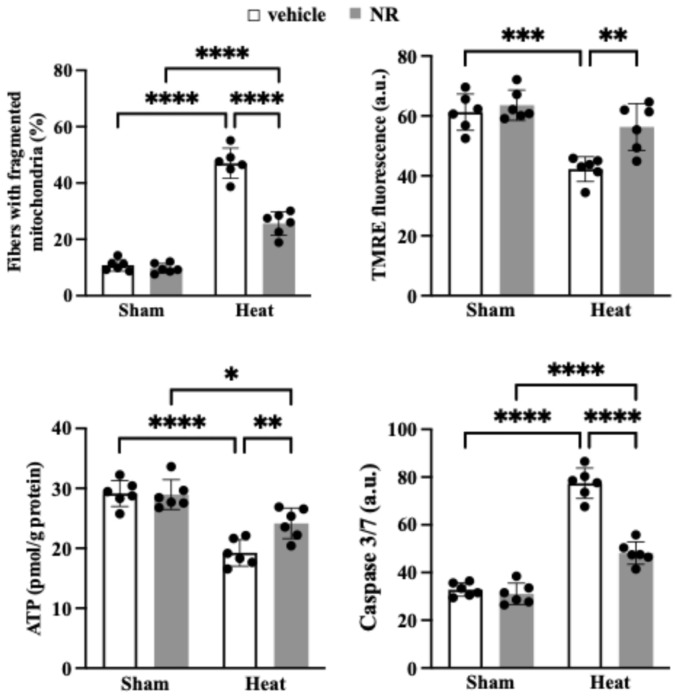
Assessment of mitochondrial impairment and apoptosis in skeletal muscle. Mitochondrial morphology, membrane potential and caspase-3/7 activation were examined in FDB muscle (Figures S2–S4) and ATP levels in gastrocnemius muscle. Data are shown as means ± SD (n = 6 mice per group). * *p* < 0.05, ** *p* < 0.01, *** *p* < 0.001, and **** *p* < 0.0001.

Mitochondrial impairment or dysfunction can cause a loss of cross-membrane proton gradient or mitochondrial membrane potential (MMP, ΔΨm) and the release of proapoptotic factors, leading to a reduced driving force for ATP production and programmed cell death (apoptosis) [16]. We have previously shown that heat stress increased mitochondrial fragmentation, reduced mitochondrial membrane potential and induced apoptosis in the skeletal muscle of male mice [9]. NR treatment mitigated these adverse effects of heat. Similar results were found in this study. Microscopy analysis revealed that heat exposure significantly increased fragmented mitochondria, reduced mitochondrial membrane potential, and induced caspase-3/7 activation, an essential event during apoptosis in FDB muscles (*p* = 0.01 to *p* < 0.0001, n = 6 per group, Figure 2 and Figures S2–S4). In addition, heat exposure also significantly decreased the levels of ATP in gastrocnemius muscles. How heat stress compromises mitochondrial ATP production is not fully known. However, there is evidence that hyperthermia can induce a number of immediate metabolic changes by increasing metabolic rates, resulting in increased turnover of ATP and depletion of energy reservoirs in cells and tissues [17]. NR treatment prevented or reduced these changes.

### 3.3. Neither Heat Exposure nor NR Alters NAD(H) Levels in Skeletal Muscle

**Figure 3:**
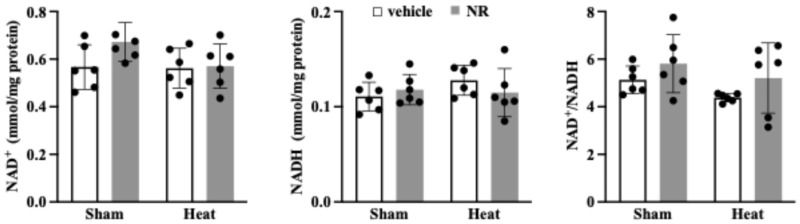
NAD(H) levels in gastrocnemius muscle. Data are shown as means ± SD (n = 6 mice per group).

We next sought to investigate whether NR treatment would alter skeletal muscle NAD(H) homeostasis. We have recently reported that NR treatment boosted NAD(H) availability and prevented heat-induced NAD^+^/NADH imbalance in mouse intestine tissues [15]. In this study, the measurement of NAD^+^ and NADH levels in gastrocnemius muscles revealed no significant effects of NR or heat exposure (*p* > 0.05, n = 6 per group, Figure 3). Similarly, no differences in the NAD^+^/NADH ratio were found between the vehicle and NR-treated or between the sham and heat-exposed mice. These results indicate that the prevention of skeletal muscle heat injury by NR did not involve an alteration in NAD(H).

### 3.4. Heat Exposure Causes Local and Systemic Mg^2+^ Depletion

**Figure 4:**
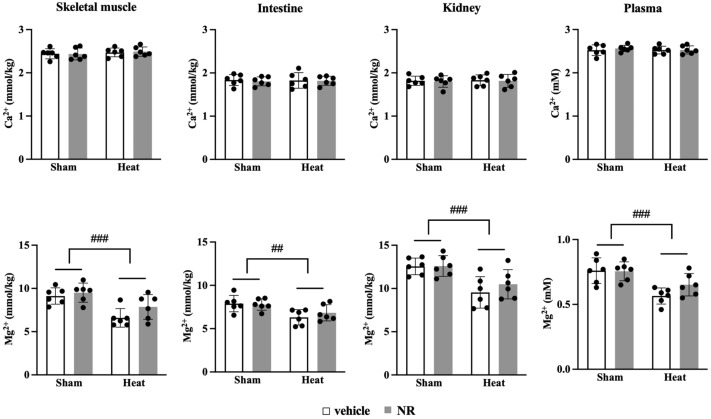
Ca^2+^ and Mg^2+^ concentrations in plasma, gastrocnemius muscle, intestine, and kidney. Data are shown as means ± SD (n = 6 mice per group). ## *p* < 0.01, and ### *p* < 0.001 for the heat effect.

Understanding the mechanisms underlying heat-induced loss of MMP may help in the development of strategies against heat injury. The evidence suggests that prolonged opening of mitochondrial permeability transition pore (PTP) leading to dissipation of MMP contributes to mitochondrial disorders in various pathological conditions, and Ca^2+^ and Mg^2+^ serve key roles in regulating PTP [18]. We have previously shown that heat exposure decreased the skeletal muscle Mg^2+^ content in male mice [9]. NR treatment reduced injury-related changes but had no effect on Mg^2+^ in the skeletal muscle. In this study, we sought to examine whether heat exposure would deplete Mg^2+^ in female mice. We measured both Ca^2+^ and Mg^2+^ concentrations in the gastrocnemius muscle, jejunum, kidneys, and plasma. The intestine and kidneys are among the most vulnerable organs to heat injury [2,19]. There were no significant differences in tissue or plasma Ca^2+^ concentrations between the sham and heat-exposed mice with or without NR treatment (Figure 4). However, Mg^2+^ concentrations in all the three organ tissues and plasma were significantly lower in the heat-exposed mice than in the sham controls (*p* = 0.001–0.0004, n = 6 per group). NR treatment had no effect on the tissue or blood Mg^2+^ concentrations. These results indicate that NR prevented skeletal muscle heat injury without affecting the Ca^2+^ or Mg^2+^ levels in the organs and plasma.

### 3.5. Heat Exposure Disrupts Mitochondrial Ca^2+^ and Mg^2+^ Homeostasis in Skeletal Muscle

**Figure 5:**
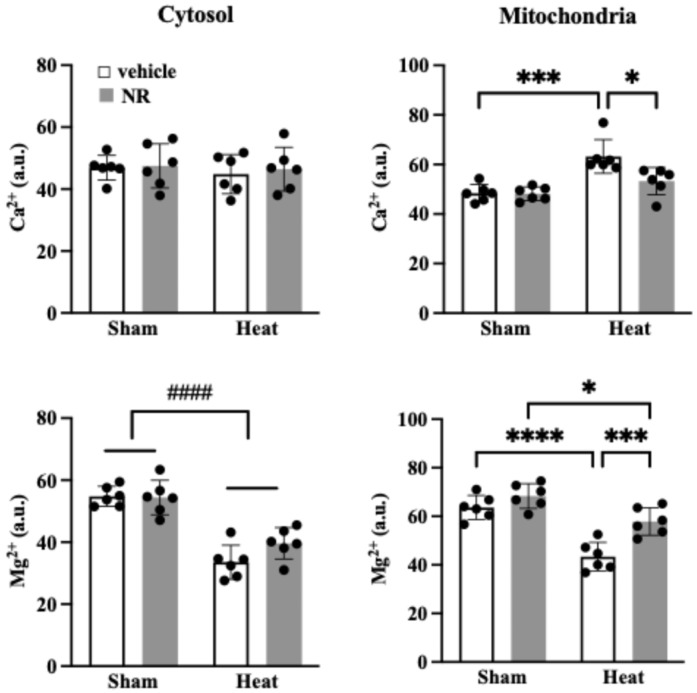
Cytosolic and mitochondrial Ca^2+^ and Mg^2+^ levels in FDB muscle. Ca^2+^ and Mg^2+^ levels were quantified based on their cytosolic and mitochondrial fluorescence (Figures S5 and S6). Data are shown as means ± SD (n = 6 mice per group). * *p* < 0.05, *** *p* < 0.001, and **** *p* < 0.0001. #### *p* < 0.0001 for the heat effect.

Using fluorescence microscopy, we further examined the Ca^2+^ and Mg^2+^ levels in the cytosol and mitochondria of FDB muscles (*p* = 0.01 to *p* < 0.0001, n = 6 per group, Figure 5, Figures S5 and S6). No effects of heat exposure or NR were detected on cytosolic Ca^2+^ levels. Heat exposure significantly increased the mitochondrial Ca^2+^ levels in the mice treated with vehicle and not in those treated with NR. In contrast, heat exposure decreased both the cytosolic and mitochondrial levels of Mg^2+^. NR treatment reduced this Mg^2+^ decline in the mitochondria and not in the cytosol. Together, these results indicate that the prevention of skeletal muscle heat injury by NR was associated with the maintenance of mitochondrial Ca^2+^ and Mg^2+^ homeostasis.

## 4. Discussion/Conclusions

**Figure 6:**
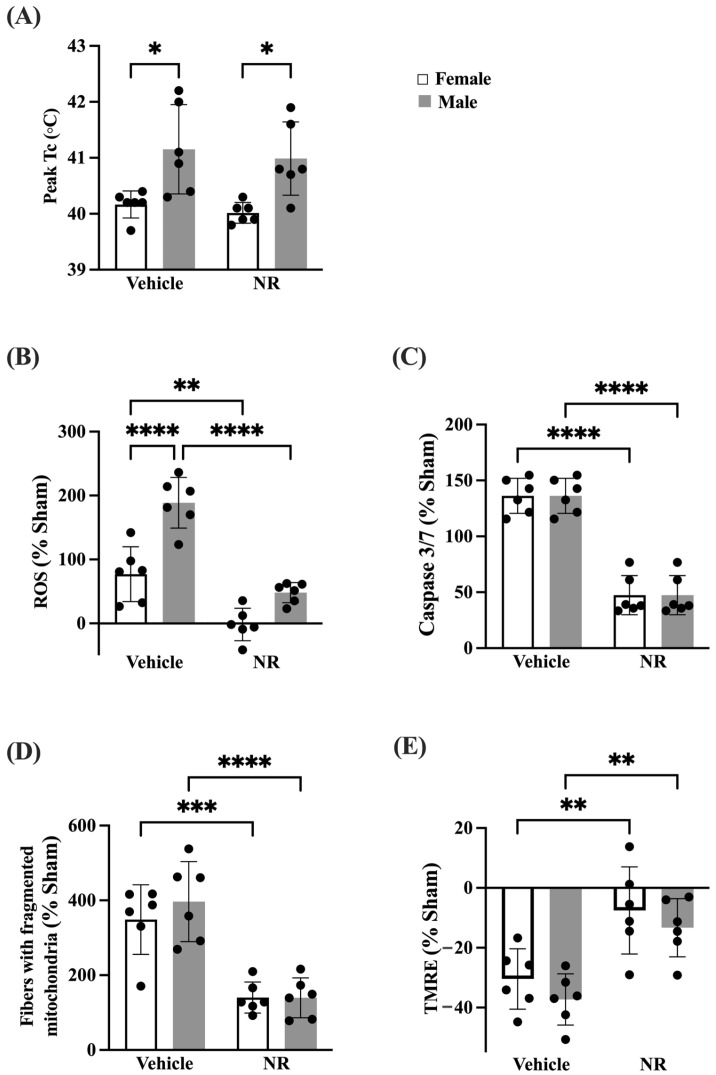
Comparison of female and male mice. Peak Tc (**A**) and FDB muscle ROS (**B**), mitochondrial fragmentation (**C**), caspase 3/7 (**D**), and mitochondrial membrane potential (**E**). Data are expressed as a percent change against a corresponding sham value. n = 6 mice per group. * *p* < 0.05, ** *p* < 0.01, *** *p* < 0.001, and **** *p* < 0.0001.

We show here that heat exposure caused inflammation, oxidative stress, apoptosis, and mitochondrial dysfunction in skeletal muscle. NR supplementation effectively protected against these changes, mirroring our previous findings in male mice. These results confirm the hypothesis that NR improves skeletal muscle heat injury in female mice. A comparison with the previously published data from male mice under identical experimental conditions [9] revealed similar patterns of heat-induced changes in the FDB muscles of both sexes, despite females exhibiting a significantly lower peak Tc during heat exposure (Figure 6A). In both sexes, heat exposure increased reactive oxygen species (ROS) production (Figure 6B), caspase 3/7 activation (Figure 6C), and mitochondrial fragmentation (Figure 6D), while reducing mitochondrial membrane potential (Figure 6E). Except for slightly lower ROS levels in females without NR, no other sex differences were observed. Crucially, NR supplementation proved equally effective in both females and males in preventing these heat-induced skeletal muscle alterations.

Interestingly, unlike our previous findings in male mice [9], NR supplementation did not increase muscle NAD^+^ in this study. Similar inconsistencies regarding NR’s effect on skeletal muscle NAD^+^ have been reported in studies using high-fat diet mouse models of type 2 diabetes, where NR improved whole-body glucose metabolism and skeletal muscle oxidative function, yet it either increased [20] or did not affect [21] the skeletal muscle NAD^+^ content. To our knowledge, this is the first study to show that NR treatment protects against muscle heat injury without increasing NAD^+^ availability. It is worth noting that heat exposure had no effect on cytosolic NAD^+^ in male mice; it reduced the mitochondrial NAD^+^ levels, which NR supplementation partially restored [9]. Similarly in this study, we found that following heat exposure, NAD^+^ levels in the skeletal muscle supernatant fraction (primarily cytosol) of female mice remained unaltered, though the mitochondrial NAD^+^ content was not measured. Of note, most NAD(H) detected in the mouse tissues is cytosolic. The intracellular distribution of NAD(H) is highly compartmentalized. Cytoplasmic and nuclear NAD(H) pools are believed to be more sensitive to changes in redox stress than the mitochondrial NAD(H) pool [22]. Collectively, these findings indicate that NR prevented mouse skeletal muscle from heat-induced injury without boosting tissue NAD^+^. Indeed, NAD^+^ precursors may have various biological activities that are independent of their conversion to NAD^+^ [23]. They can regulate the activity of certain enzymes, such as CD38 and sirtuins, and have been shown in various studies to improve cellular resilience to oxidative stress by activating antioxidant pathways. For example, NAD^+^ precursor treatment can exert an antioxidant effect in models of acute and chronic stress through the activation of mitochondrial NAD^+^-dependent sirtuins [24,25]. Oxidative stress is known to contribute to skeletal muscle heat injury [26]. Together, the preventive effect of NR might be a direct signaling result of the precursor itself, rather than solely dependent on the downstream production of NAD^+^.

Consistent with our findings in male mice [9], this study showed that NR prevented mitochondrial Ca^2+^ and Mg^2+^ homeostasis from heat-induced alterations. Elevated mitochondrial Ca^2+^ has been linked to various pathophysiological conditions, including ischemic heart disease, stroke, obesity, and chronic neurodegenerative disorders [27]. Information on stress-induced mitochondrial Mg^2+^ depletion is scarce. The mechanisms underlying heat-induced mitochondrial Ca^2+^/Mg^2+^ imbalance remain unclear. Both Ca^2+^ and Mg^2+^ are highly compartmentalized and tightly controlled [28,29,30]. There is evidence suggesting that the divalent cations Ca^2+^ and Mg^2+^ play a role in regulating mitochondrial function; excessive mitochondrial matrix Ca^2+^ is thought to drive the sustained opening of the mitochondrial permeability transition pore (PTP) [27], while Mg^2+^ prevents PTP opening [31]. Sustained PTP opening can lead to mitochondrial impairment and ultimately cell death. In this study, heat-induced MMP reduction may be an indication of PTP opening [18]. In both the male and female mouse studies, we found that NR protected mitochondrial Ca^2+^ and Mg^2+^ homeostasis, but it had no effect on tissue Mg^2+^ depletion in response to heat stress. How an NAD^+^ precursor interacts with intracellular Ca^2+^ or Mg^2+^ fluxes is not fully understood. ROS has been implicated in Ca^2+^ and Mg^2+^ regulation; excessive ROS increases Ca^2+^ release from the endoplasmic reticulum and Ca^2+^ uptake by mitochondria [32] and reduces intracellular Mg^2+^ concentration [33]. It is unclear whether NR protection of mitochondrial Ca^2+^ and Mg^2+^ homeostasis against heat stress is an indirect result of its antioxidant effect. Furthermore, in this study, reduced Mg^2+^ levels were found not only in skeletal muscle but also in internal organ tissues and plasma following heat exposure. Depletion of circulating Mg^2+^ is reportedly associated with chronic diseases [34,35]. We are unaware of previous studies examining the effects of acute heat stress on the blood or organ Mg^2+^ levels. The fact that NR had no effect on whole tissue and circulating Mg^2+^ levels suggests that the systemic Mg^2+^ loss is likely a consequence rather than a cause of heat stress.

This study has certain limitations. While this work extended our previous NAD^+^ findings in the sarcoplasm by revealing similar changes in skeletal muscle tissue, mitochondrial NAD^+^ was not measured. Therefore, unlike our male mouse study, we cannot confirm whether NR impacts mitochondrial NAD^+^ homeostasis. Additionally, estrous cycle phases were not assessed. However, we previously demonstrated no significant effect of estrous cycles on the hyperthermic response of female mice during heat exposure [13], suggesting that sex hormonal fluctuations likely did not influence the NR treatment outcomes for heat-induced skeletal muscle injury.

Our results indicate that despite a lower hyperthermic response, female mice exhibit skeletal muscle changes similar to those in male mice following heat exposure. NR treatment protects skeletal muscle against heat injury, likely through anti-oxidative stress, anti-inflammatory, and anti-apoptotic mechanisms. Unlike in males, neither heat stress nor NR affected muscle NAD^+^ homeostasis in female mice. There is no evidence that the effectiveness of NR administration in preventing skeletal muscle heat injury is influenced by sex.

## Figures and Tables

**Table 1:** Body core temperature (Tc).

	Baseline Tc, °C	Peak Tc, °C
Vehicle	36.8 ± 0.4	40.2 ± 0.2 *
NR	36.7 ± 0.6	40.0 ± 0.2 *

n = 6 per group. * *p* < 0.001 versus baseline Tc.

## Data Availability

The original contributions presented in this study are included in the Supplementary Material. Further inquiries can be directed to the corresponding author.

## Supplementary Materials

The following supporting information can be downloaded at https://www.mdpi.com/article/10.3390/muscles5020044/s1, Figure S1: Representative images of DHE fluorescence in FDB muscles.; Figure S2: Representative images of MitoTracker-stained mitochondria in FDB muscles; Figure S3: Representative images of TMRM fluorescence in FDB muscles; Figure S4: Representative images of caspase-3/7 green fluorescence in FDB muscles; Figure S5: Representative images showing fluorescence detection of Ca^2+^ in FDB muscles. A. Cytosolic Fura-2 fluorescence. B. Mitochondrial colocalization of Rhod-2 and MitoTracker fluorescence; Figure S6: Representative images showing detection of Mg^2+^ in FDB muscles by Mag-Fluo-4 AM staining. A. Cytosolic Mag-Fluo-4 AM fluorescence. B. Mitochondrial colocalization of Mag-Fluo-4 AM and MitoTracker fluorescence.
