# Supplementary material for: Nicotinamide Riboside Supplementation Protects Against Heat-Induced Skeletal Muscle Injury in Female Mice
Source: Muscles. 2026 Jun 15;5(2):44. doi: 10.3390/muscles5020044 (PMC13304672; doi:10.3390/muscles5020044)

Supplementary material

Figure S1. Representative images of DHE fluorescence in FDB muscles.

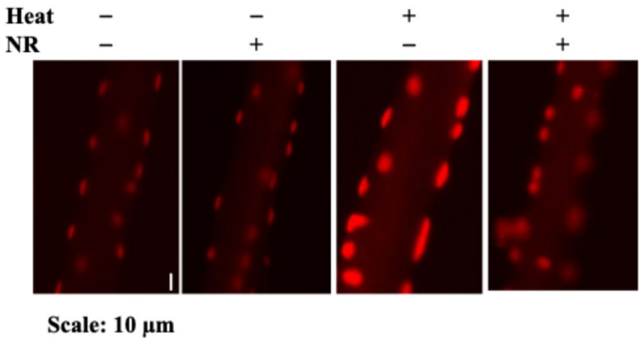

Figure S2. Representative images of MitoTracker-stained mitochondria in FDB muscles.

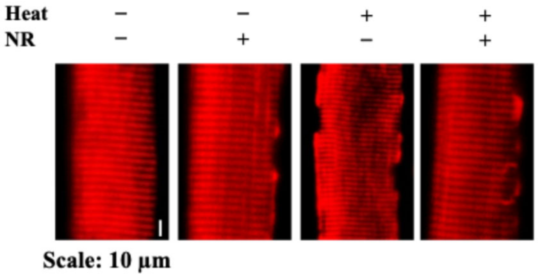

Figure S3. Representative images of TMRM fluorescence in FDB muscles.

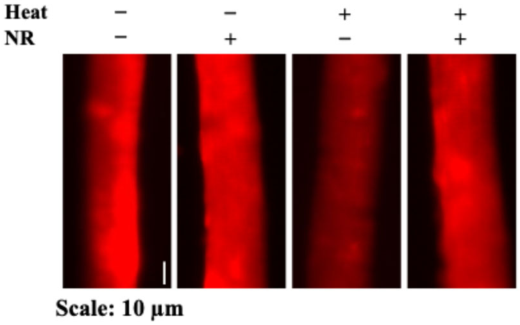

Figure S4. Representative images of caspase-3/7 green fluorescence in FDB muscles.

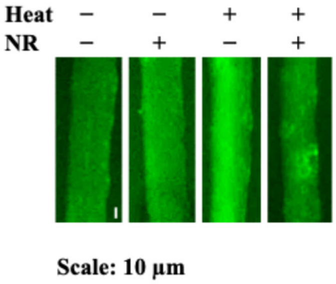

Figure S5. Representative images showing fluorescence detection of Ca^{2+} in FDB muscles. A. Cytosolic Fura-2 ~~fluorecence~~fluorescence. B. Mitochondrial colocalization of Rhod-2 and MitoTracker fluorescence.

(A)

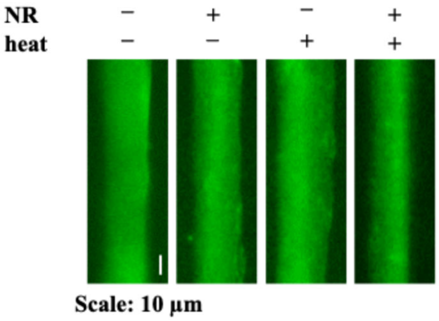

(B)

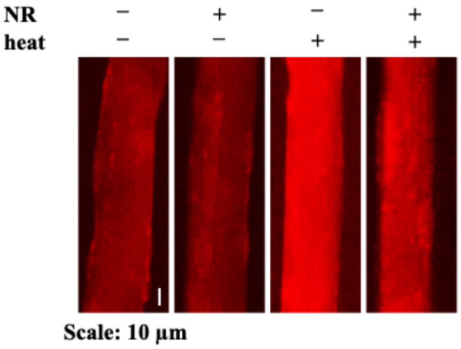

Figure S6. Representative images showing detection of Mg^{2+} in FDB muscles by Mag-Fluo-4 AM staining. A. Cytosolic Mag-Fluo-4 AM fluorescence. B. Mitochondrial colocalization of Mag-Fluo-4 AM and MitoTracker fluorescence.

(A)

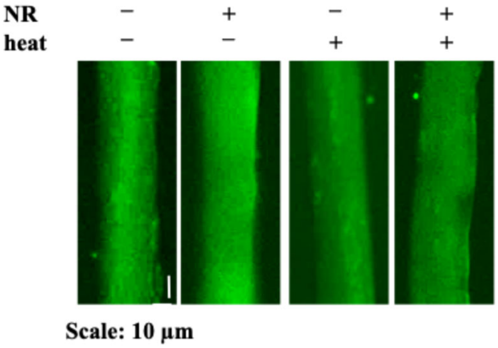

(B)

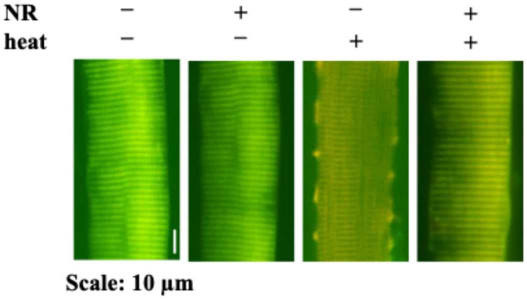

Supplement: Supplementary file 1 [file muscles-05-00044-s001.zip › muscles-4234660-supplementary.pdf]
